# Extensive Spinal Hemangioma Associated With Cutaneous Nevus in the Same Metamere: An Unusual Case of Paraplegia in the Peripartum Period

**DOI:** 10.7759/cureus.54673

**Published:** 2024-02-22

**Authors:** Rogelio D Flores Reyes

**Affiliations:** 1 Neurosurgery, Mexican Social Security Institute, Veracruz, MEX

**Keywords:** nevus flammeus, angiomatosis, compressive myelopathy, peripartum, spinal angioma, cavernous hemangioma

## Abstract

Cavernous hemangiomas occur most commonly in the cerebral hemispheres but can involve any part of the neuroaxis, including the spine. Very rare cases of spinal angiomas are associated with a skin lesion in the same metameric segment. This condition, known as segmental neurovascular syndrome or Cobb syndrome, was first described in 1915. We report a rare case of segmental neurovascular syndrome with extensive cervical and thoracic lumbar involvement expressed as peripartum spinal cord compression syndrome. A 37-year-old female with a cutaneous nevus from the C7 dermatome to the L3 dermatome experienced pelvic limb paralysis 48 hours after giving birth to a healthy newborn by cesarean section. Magnetic resonance imaging (MRI) revealed an enhancing extensive epidural mass from C7 to T7 and subsequently from T10 to L3. Histopathology confirmed a spinal cavernous hemangioma. Although rare, segmental neurovascular syndrome must be considered in patients with cutaneous angioma and radiculopathy or myelopathy. Early diagnosis can lead to curative surgical treatment and more favorable outcomes.

## Introduction

Spinal angiomas represent 12% of all vascular pathologies of the spinal cord and 4% of lesions in the epidural space [1]. Spinal cavernous hemangiomas most commonly arise from the vertebral body. They are often diagnosed as incidental findings or as clinical findings in patients with compressive myelopathy caused by the lesion extending into the epidural space and compressing the spinal cord. Clinical manifestations typically progress slowly as spinal cord compressive syndrome or radiculopathy. The sudden onset of symptoms is often secondary to the enlargement of the lesion caused by intralesional hemorrhage, thrombosis, increased vascularization caused by hormonal effects, or mechanical venous occlusion [2]. Very rare cases of spinal angiomas are associated with a skin lesion in the same metameric segment, known as segmental neurovascular syndrome or Cobb syndrome. This non-hereditary neurocutaneous disease is characterized by spinal vascular malformations associated with cutaneous vascular malformation in the same metameric segment. Skin lesions may occur anywhere in the dermatome, from the midline of the back to the abdomen [3]. Since the first case of Cobb syndrome was described in 1915, only 40 cases have been reported in the international literature, 20 of which involved children [3]. In patients with myelopathy (or less commonly radiculopathy) and a congenital nevus, magnetic resonance imaging (MRI) can identify the location and characteristics of a spinal vascular malformation and aid in diagnosis. The clinical finding of this vascular-related pathology in the peripartum period clearly states an important influence from hormonal and hemodynamic factors related to the pregnancy that can be directly related to an earlier clinical manifestation.

## Case presentation

A 37-year-old female in the postpartum period was referred to our hospital. Her history included paresthesia in the pelvic limbs for two months, with progression to paraparesis for one-and-a-half months. Eleven days prior to her admission, she underwent a cesarean section in another hospital due to prolonged labor. Approximately 48 hours after the cesarean section, the paresis worsened to paraplegia. Upon her admission, two purplish-red, macular lesions with well-defined borders were observed in the thoracic and posterior lumbar regions. A gynecological examination revealed inadequate uterine involution, indicating possible hysterorrhaphy dehiscence. She was admitted to the operating room, where uteroperitoneal fistula was confirmed. A hysterectomy plus a right salpingo-oophorectomy was performed without incident. Four days after the gynecological procedure, she underwent a laminoplasty of T12-L2 and a partial resection of the lesion, where findings indicated a highly vascularized extradural lesion in the right dorsolateral region of the spinal canal. Histopathology revealed a cavernous hemangioma.

**Figure 1:**
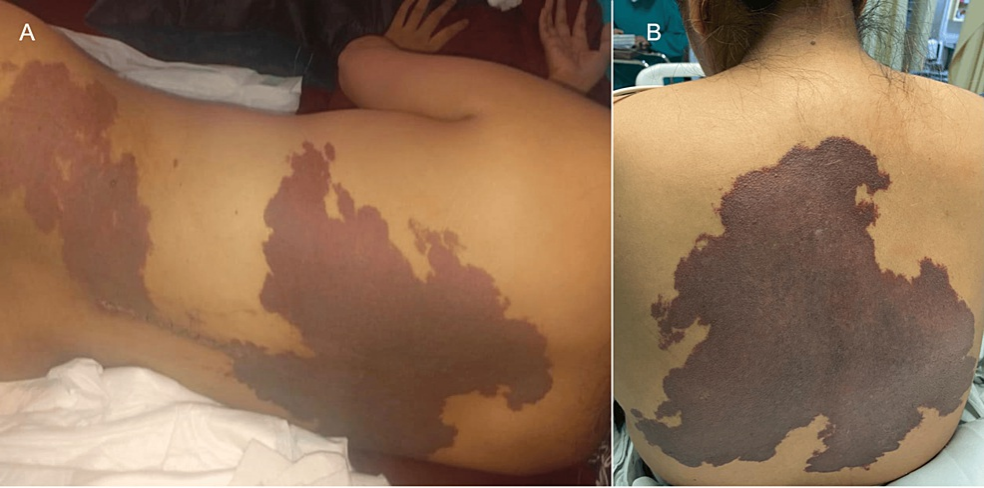
Cutaneous nevus The patient with purplish-red cutaneous nevus exhibiting monomorphic macules with well-defined margins located in the (A) dorsal thoracic and lumbar regions and (B) dorsal region of the chest

The examination findings showed muscle strength of five out of five for C5-T1 and zero out of five for L2-S1, deep bicipital and supinator tendon reflexes of ++/++++, and patellar reflex and bilateral Achilles reflexes of +++/++++, accompanied by exhaustible clonus reflex and diminished tone. Sensitivity from C1 to T8 was preserved, and pain and temperature sensitivity from T9 to the distal dermatome decreased. Local mechanical pain was present but not radicular or myelopathic pain. There was no alteration of the urinary sphincter. Physical inspection revealed the presence of two purplish-red, macular lesions with well-defined borders in the thoracic and posterior regions (Figure 1A, 1B).

**Figure 2:**
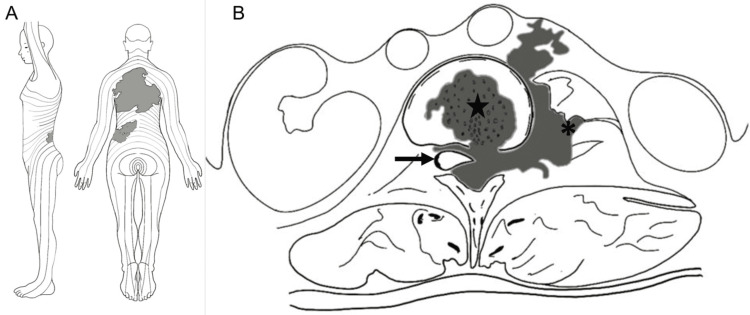
Diagram showing the extension of the cutaneous nevus (A) The extension of the cutaneous nevus from the C7 dermatome to the L3 dermatome (left lateral and posterior views). (B) A diagram of an axial section at L2 representing the displacement of the conus medullaris (arrow), the involvement of the vertebral body (star), and extension through the left neuroforamen to reach the ipsilateral psoas muscle (asterisk). Image credit: Rogelio D. Flores Reyes Jr.

The cutaneous nevus extended from the C7 dermatome to the L3 dermatome, as represented in the diagram in Figure 2A, 2B.

Imaging findings

**Figure 3:**
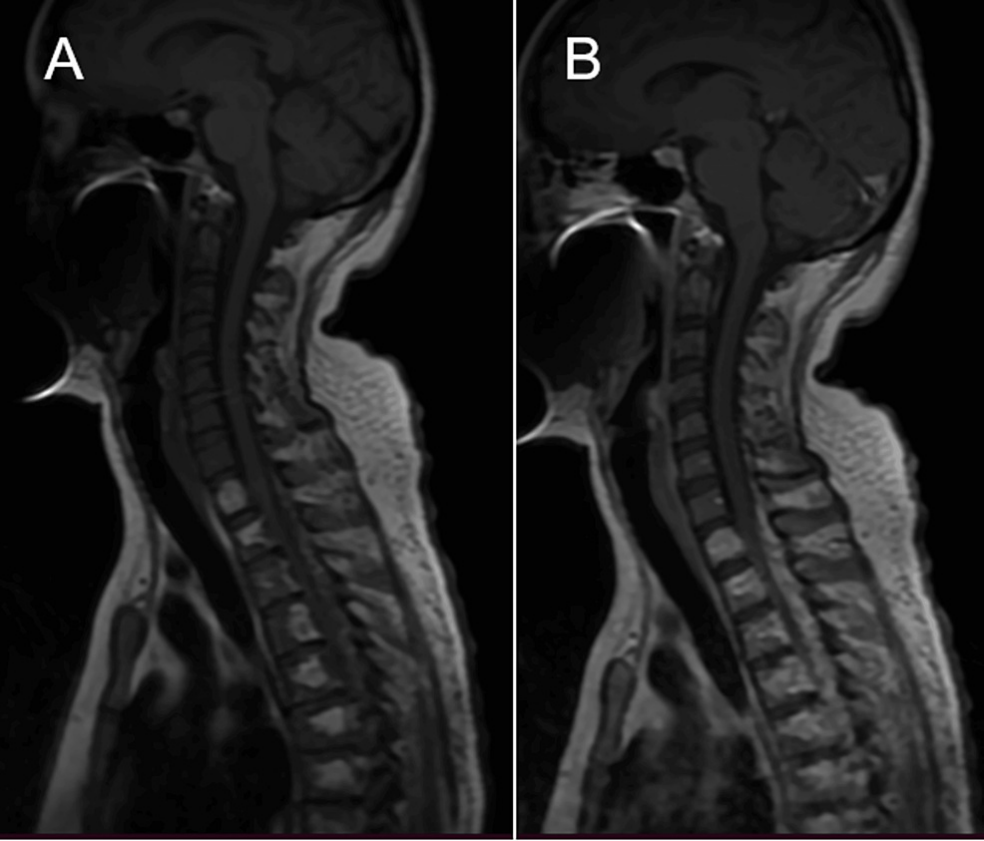
Sagittal magnetic resonance imaging of the cervical and upper thoracic spinal cord (A) T1-weighted image showing predominantly hyperintense extradural mass at the posterior margin of the spinal cord, caudally from C7. (B) T1 using a gadolinium-based contrast agent showing the same lesion with homogeneous contrast enhancement and the involvement of vertebral bodies from C8

**Figure 4:**
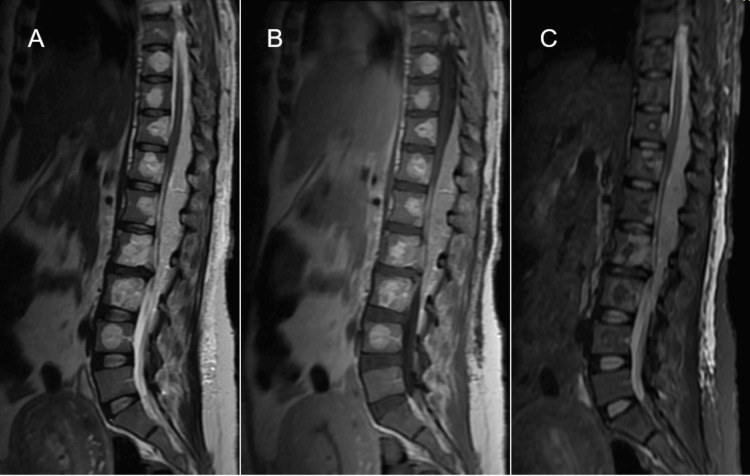
Sagittal thoracolumbar MRI (A) T2-weighted sequence showing hyperintense epidural mass extending from T10 to L3 and invading the vertebral bodies from T9 to L4, causing the effacement of the thecal sac and the compression of the spinal cord. (B) T1-weighted image showing hyperintense lesion in relation to the spinal cord. (C) STIR-weighted MRI showing a hyperintense image with respect to the spinal cord and a hypointense image with respect to the cerebrospinal fluid MRI, magnetic resonance imaging; STIR, short tau inversion recovery

**Figure 5:**
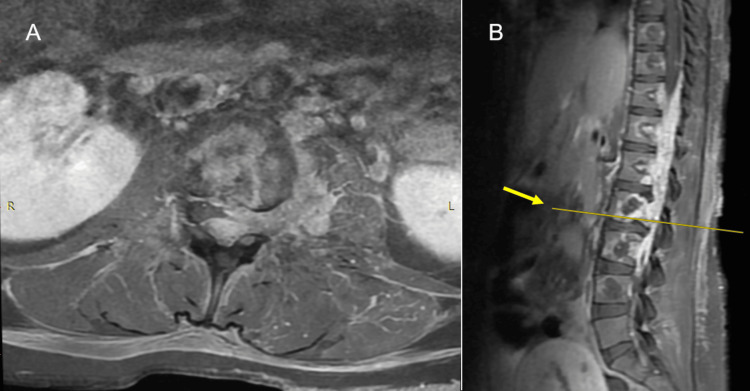
Axial and sagittal MRI at L2 level (A) Axial T1-weighted MRI using gadolinium-based contrast agent showing the involvement of the vertebral body, an epidural occupational lesion causing displacement to the right of the cauda equina, and extension to the left neuroforamen L2-L3 with the involvement of ipsilateral psoas muscle. (B) Sagittal T1-weighted MRI using gadolinium-based contrast agent showing the extension of the lesion into the craniocaudal axis from its lower portion from T10 to L3 (arrow and line indicate L2 level) MRI: magnetic resonance imaging

**Figure 6:**
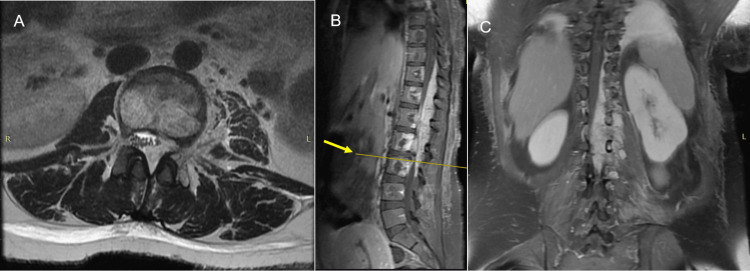
MRI showing thoracolumbar extension (A) Axial T2-weighted MRI at L3 level showing the displacement of the nerve roots of the cauda equina. (B) Sagittal T1-weighted MRI showing the hyperintense behavior of the lesion (arrow and line indicate L3 level). (C) Same-level coronal T1-weighted MRI using gadolinium-based contrast agent showing the left predominance of the lesion with extension toward the ipsilateral neuroforamen MRI: magnetic resonance imaging

The MRI of the cervical and thoracolumbar spine was performed using a gadolinium-based contrast agent. The MRI revealed an extraspinal and intraspinal extradural lesion. The T1-weighted sequence showed a predominantly hyperintense extradural lesion of lobulated morphology with well-defined margins, located predominantly at the posterior, left lateral margin and to a lesser extent anterior to the spinal canal with extension from C7 to T7 and subsequently from T10 to L3. The lesion compressed the thecal sac and displaced the medullary cord. In addition, the extension of the lesion was observed at the foraminal level from T11 to L2 on the left side with the invasion of vertebral bodies from C8 to L4. The extension of the lesion into the fatty tissue and left iliac psoas muscle also was observed. Short tau inversion recovery (STIR)-weighted MRI showed a hyperintense image with respect to the spinal cord and a hypointense image with respect to the cerebrospinal fluid (Figures 3-6).

Surgical procedure and findings

A posterior approach guided by fluoroscopy was performed via incision in the posterior midline. Paraspinal muscles were dissected from the laminae of T12-L2. The bony laminae of T12-L2 were cut with the use of a craniotome. We explored the epidural space, observing a dark purple mass on the dorsolateral side, predominantly on the left, vascularized, with the thinning of the dura mater. A partial tumor resection at that level was performed. Bony laminae were replaced and affixed in position.

Histopathological findings

**Figure 7:**
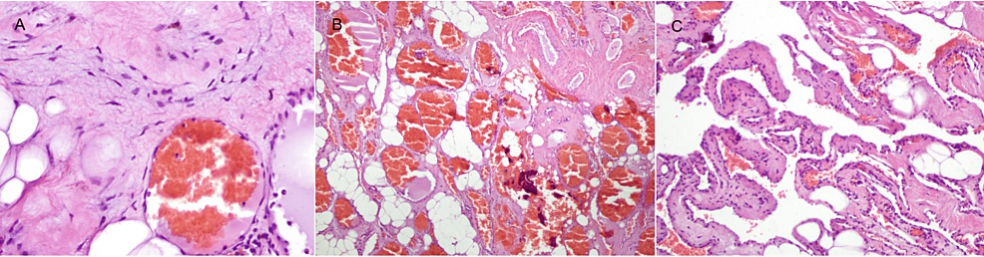
Photomicrograph (hematoxylin and eosin staining) (A) Original magnification: ×10. (B) A magnification of ×20 showing venous vessels with simple flat endothelium and without atypia. (C) A magnification of ×5 showing nutrient arterial vessels in the upper right corner and congestive venous vessels in irregular compact groups alternating with dystrophic calcifications

Histopathology confirmed a cavernous hemangioma. In the histological section, the epidural lesion was composed of congested venous vessels in compact irregular groups alternating with dystrophic calcifications. The vessel walls had irregular thickness with anastomosed lumens and without obvious elastic fibers. The endothelium showed no evidence of atypia (Figure 7).

## Discussion

Spinal cavernous hemangiomas are vascular malformations with a predilection for the thoracic spine. MRI is diagnostic in these cases, and the total removal of the mass is optimal [1]. On average, two-and-a-half vertebral segments are involved in each case, suggesting that epidural cavernous hemangiomas are more likely to grow laterally than longitudinally [4]. In this case, both lateral and longitudinal extensions were observed. A pure epidural location, defined as 90% of the tumor volume in the epidural space, is unusual [5]. Even more unusual is the cervical, thoracic, and lumbar extension described in this case.

Intraspinal vascular lesions can be categorized as vascular tumors (hemangioblastoma and cavernous malformation) and arteriovenous malformations, as well as epidural and intradural lesions [4]. A key MRI finding in suspected arteriovenous malformations is the presence of intense-flow voids due to the high-flow velocity of the vascular structures, which can be a key piece of information in the differential diagnosis of these lesions. Acute hemorrhage often leads to neurologic deficit, requiring urgent decompressive surgery. The degree of functional recovery often depends on the location of the bleeding (i.e., epidural or intramedullary). In most cases, epidural bleeding has a better prognosis as it does not involve direct damage to neural tissue.

Since 1915, when Cobb first described a spinal hemangioma associated with paraplegia, isolated cases of spinal vascular malformations associated with cutaneous vascular lesions have been reported. The historically low frequency of cases may be due to the lack of clinical expression of quiescent lesions. The vascular skin nevus found with Cobb syndrome is accompanied by a large variety of vascular pathologies. Intraspinal lesions are usually arteriovenous malformations (high-flow lesions) and rarely angiomas (low-flow lesions) [6].

Although the location of spinal cavernous hemangiomas is usually limited to two or three vertebral segments at the thoracic level, in this case, we observed extensive longitudinal spread at the cervical, thoracic, and lumbar levels, as well as a tendency to invade the vertebral bodies and paravertebral region to reach the left iliopsoas muscle. Although malignancy was suspected, cellular atypia was not observed in the histopathological study. The lesion distribution thus was more likely caused by its association with a cutaneous nevus, suggesting that the spinal lesion was present from birth, which was further indicative of segmental vascular syndrome.

Spinal cavernous hemangiomas usually originate from the vertebral bodies, with occasional secondary extension into the extradural space. The affected vertebrae have coarse trabeculae, which may be a critical finding for differentiating epidural cavernous hemangiomas of vertebral origin from foraminal nerve sheath tumors [7]. The pattern of involved vertebrae has been described as vertical striations in sagittal reformatted images and as a honeycomb pattern in transverse images [7]. MRI typically delineates a well-circumscribed lobulated lesion: T1-weighted imaging demonstrates a homogeneously isointense lesion, and T2-weighted imaging (T2WI) shows a hyperintense mass slightly less intense than cerebrospinal fluid. Uniform enhancement can be observed in gadolinium-enhanced, T1-weighted MRI [1]. Similar findings were observed in this case, although the T1-weighted sequence revealed a hyperintense image (Figure 4B).

The differential diagnosis of this condition should include other epidural tumors, such as lymphomas, metastatic tumors, meningiomas, or neurinomas-neurofibromas [8]. When cavernous hemangiomas extend through the foramen, they do not enlarge the neuroforamen as much as a similarly sized schwannoma or neurofibroma, and they often have an irregular or lobulated contour with strong enhancement [4]. Lymphomas usually appear as isointense on T2-weighted images and exhibit less frequent paravertebral extension and intervertebral neural foraminal widening. An angiolipoma is typically hyperintense on T1-weighted images because of its fat content, which is typically absent in a cavernous hemangioma [4]. An MRI with fat-suppression sequences should therefore be performed to rule out this tumor type [8]. Extension through the intervertebral foramen region creates a dumbbell shape on axial magnetic resonance [9], which can confuse the diagnosis with lesions such as meningioma and schwannoma [2]. In neurogenic tumors, however, a smooth rather than lobulated contour and frequent cystic changes could aid in the differential diagnosis of cavernous hemangiomas [9]. Hemangiomas in the intramedullary region exhibit a mixed signal with a hypointense hemosiderin ring on T2WI due to previous bleeding [2]. Epidural cavernous hemangiomas typically do not present in this way, likely due to the absence of a blood-spinal barrier in the epidural space, which favors the faster removal of hemosiderin.

In pregnancy, multiple mechanisms can promote the clinical expression of these lesions, such as direct stimulation through growth factors or hormones or venous congestion caused by changes in the venous flow linked to the growth of the gravid uterus. In our case, symptoms occurred and worsened in the last trimester of pregnancy, which may be related to the increase in estrogen at this stage; however, other processes that could explain an acute onset or worsening must be considered.

The treatment of this type of lesion consists of complete surgical resection through a posterior spinal approach, in most cases a laminectomy. In this case, given the extension of the lesion from C7 to L3, the instrumentation of a large extension of the spine was necessary. The risks and benefits of a complete surgical resection versus a more conservative surgical management were evaluated. A three-level laminoplasty was chosen with the objective of relieving pressure in the site of greatest spinal cord compression and sending the specimen to pathology for the histopathological identification of the lesion. As our patient had experienced complications during a recent hysterotomy, a less aggressive surgery was chosen to decrease postoperative complications, such as delayed wound healing and pulmonary venous thromboembolism, and to alleviate spinal compression and improve the strength of the lower extremities.

Another consideration during surgery is to perform a careful dural opening to avoid injuring vascular structures that could be adhered to the dural plane, which could increase the surgical time or risk injury due to ischemia at the spinal cord level. Coagulation in the first instance of venous drainage can increase blood congestion and thus intraoperative bleeding, so nutrient arteries must first be identified with the aim of reducing blood flow, before proceeding to coagulate venous vessels.

## Conclusions

Although the incidence of cavernous hemangioma is rare, it should be included among diagnostic possibilities when addressing an extradural soft tissue mass. The choice and timing of the surgical procedure depend on the patient's clinical presentation. In a patient with acute compressive myelopathy with acute paraplegia, urgent decompression must be performed through a laminectomy.
